# Cardiovascular toxicity induced by immunotherapy in non-small cell lung cancer: a systematic review and meta-analysis of observational studies

**DOI:** 10.3389/fonc.2025.1528950

**Published:** 2025-02-24

**Authors:** Josep Sabaté-Ortega, Eduard Teixidor-Vilà, Èlia Sais, Alejandro Hernandez-Martínez, Claudia Montañés-Ferrer, Núria Coma, Emma Polonio-Alcalá, Victor Pineda, Joaquim Bosch-Barrera

**Affiliations:** ^1^ Department of Medical Oncology, Catalan Institute of Oncology, Dr. Josep Trueta University Hospital, Girona, Spain; ^2^ Precision Oncology Group (OncoGIR-Pro), Girona Biomedical Research Institute (IDIBGI-CERCA), Salt, Spain; ^3^ Department of Medical Sciences, Medical School, University of Girona, Girona, Spain; ^4^ Cardiology Department, Dr. Josep Trueta University Hospital, Girona, Spain; ^5^ Radiology Department, Diagnostic Imaging Institute, Dr. Josep Trueta University Hospital, Girona, Spain

**Keywords:** lung cancer, immunotherapy, immune checkpoint inhibitors, cardiovascular events, prevalence

## Abstract

**Background:**

Immune checkpoint inhibitors (ICIs), an immunotherapy used in cancer treatment, are associated with potential cardiovascular (CV) toxicity. Monitoring CV issues in non-small cell lung cancer (NSCLC) patients is challenging due to their lower incidence and diversity. Hence, enhancing our understanding of CV toxicities in patients receiving ICIs is required to improve their quality of life and survival. Hence, the main objective of this study is the evaluation of CV side effects in ICI-treated NSCLC patients by assessing the prevalence and hazard of CV events.

**Methods:**

A systematic review was conducted to identify relevant studies, up to November 21st, 2023. A meta-analysis was performed to examine the data extracted from the selected studies. The random-effects model was applied to account for heterogeneity among studies, reporting results as prevalence rates and hazard ratios (HR) alongside their corresponding 95% confidence intervals (CI). Studies meeting inclusion criteria were selected and outcomes were assessed through qualitative analysis.

**Results:**

Twelve observational studies using Real world Data were included, encompassing 23,621 patients with NSCLC. Our findings indicated that patients treated with ICIs exhibited a 3% prevalence of CV events and a significantly higher hazard (HR = 1.78 (95% CI: 1.46, 2.17); p < 0.00001; I2 = 72%) compared to patients treated with other drugs.

**Conclusions:**

The treatment with ICIs caused a higher rate of CV events compared to non-ICI treatments. Nevertheless, further research is required to elucidate the underlying mechanisms and implications for patient care. This calls for continued research efforts to optimize the cardiovascular health of patients undergoing immunotherapy for lung cancer.

## 1 Introduction

Patients with lung cancer (LC), particularly those presenting with advanced or metastatic stages of the disease, have long endured high rates of morbidity and mortality (1). Histologically, LC is classified into two subtypes: small-cell lung cancer (SCLC) and non-small cell lung cancer (NSCLC). While NSCLC is more prevalent, accounting for 85% of cases, SCLC, although less common (15%), exhibits a poorer prognosis (2). First-line treatment for SCLC primarily involves chemotherapy or radiotherapy, whereas NSCLC treatment includes surgery, chemotherapy, radiotherapy, and targeted therapy (3). However, the treatment landscape for lung cancer has significantly transformed with the emergence of immune checkpoint inhibitors (ICIs).

Immune checkpoints are molecules that play a crucial role in regulating the immune response, maintaining tolerance and preventing the immune system from attacking healthy cells (3). The ICIs are mainly composed of monoclonal antibodies targeting specific checkpoint proteins, such as CTLA-4, PD-1, or PD-L1 (4). The block of this axis allows the recognition and the elimination of cancer cells (4). Drugs like atezolizumab, durvalumab, ipilimumab, nivolumab, and pembrolizumab have shown great potential in improving the outcomes of patients, demonstrating remarkable long-term survival benefits for patients with both NSCLC and SCLC, alone or in combination with chemotherapy, surgery, or radiotherapy (5).

Despite their efficacy, ICIs can also induce undesirable immune-related adverse events (irAE), including rare but potentially life-threatening cardiovascular (CV) complications (6). Therefore, growing evidence from case reports, case series, and cohort studies have increased awareness of the unexpected toxic effects on the heart associated with ICI therapy. Potential defects in cardiac conduction and myocyte function leading to arrhythmias, peri- or myocarditis, heart failure and sudden cardiac arrest have been described, even though initial trials did not specifically address ICI impact on myocardial function (7). Additionally, higher risk of venous thromboembolism (VTE) have been described during ICI treatment, with varying incidence rates influenced by type of ICI, the cancer being treated (8), the concurrence of platinum-based chemotherapy and radiation therapy (8, 9), female sex, and African-American ethnicity (8–11). Although efforts are underway to define the VTE risk associated with novel therapies, the relation between cancer immunotherapy and thrombosis is not fully comprehended and, in addition, existing studies have yielded conflicting results (9, 12–14).

Cardio-oncology is a subspecialty of cardiology that focuses on preventing and treating cardiac side effects. Given the widespread use of ICIs and their expected increase in clinical practice over the next years, cooperation among the fields of cardiology, oncology, and immunology is required. The comprehension of ICI-induced CV adverse events will have a significant impact on patient’s quality of life and survival (15).

Therefore, the main aim of this study was to evaluate the prevalence of CV events and, ultimately, to the improvement of patient outcome.

## 2 Methods

### 2.1 Search strategy and databases

A systematic review was conducted following the guidelines of the Preferred Reporting Items for Systematic Reviews and Meta-analyses (PRISMA) (9). The systemic literature search was performed using Pubmed/Medline, Cochrane Trial Register, and Google Scholar from their inception to 21st November 2023. The following terms were used: (“ICI” OR “immune checkpoint inhibitor*” OR “PD-1 inhibitor*” OR “PDL-1 inhibitor*” OR “CTLA-4 inhibitor*” OR “programmed death 1 inhibitor*” OR “programmed death ligand 1 inhibitor*” OR “cytotoxic T-lymphocyte-associated protein 4 inhibitors*” OR “Atezolizumab” OR “Avelumab” OR “Nivolumab” OR “Durvalumab” OR “Ipilimumab” OR “Pembrolizumab” OR “Pidilizumab” OR “Tremelimumab” OR “Spartalizumab” OR “Cemiplimab” OR “Sintilimab” OR “Tislelizumab” OR “Toripalimab” OR “Camrelizumab”) AND (“lung cancer” OR “lung neoplasms” OR “NSCLC” OR “SCLC”) AND (“cardi* toxicity” OR “cardiac events” OR “MACE” OR “cardiomyopathy” OR “Myocarditis” OR “heart failure” OR “pericarditis” OR “arrhythmia” OR “Myocardial Infarction”).

### 2.2 Study selection criteria

Studies were selected if they followed this PECOS: P (Patients): patients with LC; E (Exposure): ICIs or ICIs with non-ICI therapies; C (Control): non-ICI therapies; O (Outcomes): prevalence and hazard ratio of CV; S (Studies): observational studies.

### 2.3 Data extraction and quality assessment

Two reviewers screened the electronic databases. Studies were exported to EndNote Reference Library version 20.0.1 (Clarivate Analytics, London, UK) and duplicate articles were removed. Two researchers entered the data extracted from the selected studies on a computer spreadsheet. Quality assessment and bias assessment were evaluated using the New Ottawa Scale (NOS) score for observational studies and the Cochrane Collaboration Tool for clinical trials. A NOS score of 1-5 was considered a high bias risk, 6-7 was moderate, and a score >7 indicated a low bias risk.

### 2.4 Statistical Analysis

Statistical analysis was conducted using the software Review Manager (version 5.4.1). The effect size risk ratio (RR) and odds ratio (OR) along with their 95% confidence intervals (CI) were determined. The data from studies were pooled using a random effects model when heterogeneity was observed. The Chi-square test was performed to assess any differences among the subgroups. Sensitivity analysis was evaluated to determine if any individual study was driving the results and to explore reasons for high heterogeneity. As per the Cochrane Handbook, the scale for heterogeneity was considered as follows: I2 = 25–60% – moderate; 50–90% – substantial; 75–100% – considerable heterogeneity, and P < 0.1 indicated significant heterogeneity (17). Analysis of the results was performed by calculating the inverse variance (IV) or hazards ratio (HR) with their respective 95% CI. Prevalence was calculated from the raw data. This, together with other extracted information, was used to find standard errors (SE) using the following formula:


$$\text{SE= }\sqrt{\frac{\text{p × (1-p)}}{\text{n}}}$$

Where “p” and “n” indicated the prevalence and the number of patients in the experimental group, respectively. The prevalence and SE of each study were then input in the Review Manager through the inverse variance method to compute pooled prevalence along with a 95% CI. Levels of significance were considered at p < 0.05 for all analyses (16).

## 3 Results

### 3.1 Literature search results

**Figure 1:**
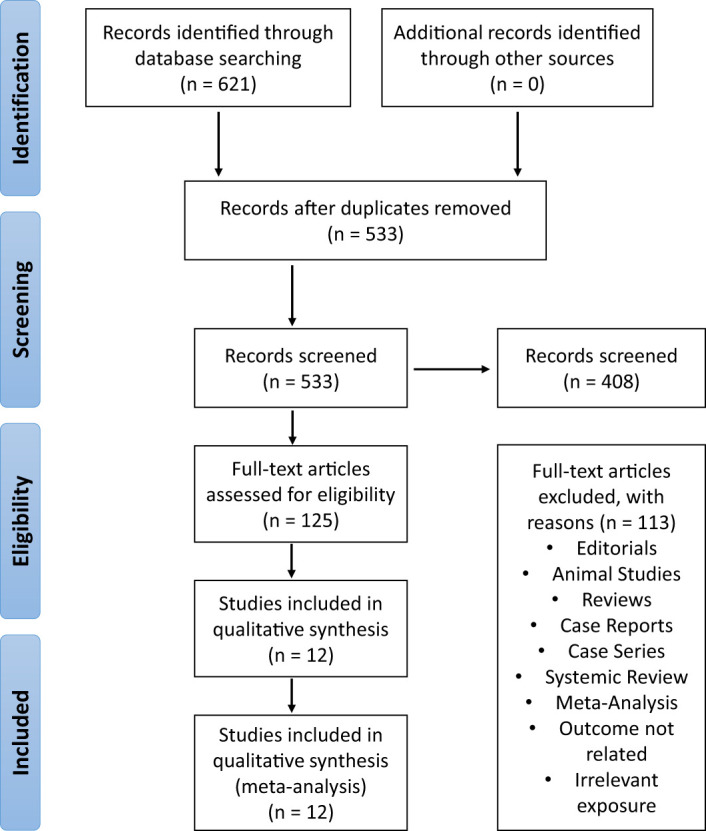
The stepwise process from initial study identification, screening, determination of eligibility, and final study inclusion, as illustrated in the PRISMA flow chart of included and excluded studies, resulted in the selection of twelve observational studies.

The initial literature search was conducted across three electronic databases (PubMed, Cochrane Central, and Google Scholar), identifying a total of 621 studies. After reviewing and reading the titles and abstracts, 125 studies were included for further analysis. Out of these, 12 observational studies that used Real World Data (RWD) were assessed for eligibility. **Figure 1** summarizes the results of the literature research.

### 3.2 Study characteristics

The twelve observational studies consisted of three prospective studies and nine retrospective studies. Five studies were conducted in Europe, four studies in America, and three studies in Asia. The patient population of these twelve studies was 23,621 and their mean age of patients was 66.06 years. The clinical and demographic details of the studied included in this meta-analysis are provided in **Table 1** (9, 17–27).

**Table 1:** Study characteristics of observational studies evaluating cardiovascular toxicity in patients with NSCLC.

AUTHOR	COUNTRY	STUDY DESIGN	MEAN AGE (YEARS)	SEX	SMOKING STATUS AND BASELINE COMORBILITIES(^*^)	HISTOLOGY	STAGE	INTERVENTION	COMPARATOR	MEAN TIME TO ONSET	CV ADVERSE EVENTS
**Canale et al.** (2020) (17)	Italy	Retrospective cohort study	70	**Total** n=60 **Male** n=36 **Female** n=24	-	**NSCLC** (65% ADK, 28% SCC)	Stage IIIB - IV	**ICI** (nivolumab/ pembrolizumab n= 60	**Non-ICI** n= 60	5.7 weeks	**Pericardial effusion** ICI: n= 4/60 (6.7%) Non-ICI: n= 2/60 (3.3%)
**Divisi et al.** (2021) (18)	Italy	Retrospective observational study	68.9	**Total** n=63 **Male** n=47 **Female** n=16	**Smoking status** *Current* n=40 *Former* n=17 *Never* n=6	**NSCLC** (66.7% ADK, 33.3% SCC)	Stage IIIB – IV	**3 groups:** **ICI (pembrolizumab** n=30 **Sequential-Chemo+ICI** **(pembrolizumab/nivolumab/atezolizumab)** n= 20 **Concomitant/sequential Chemoradiotherapy- ICI** **After radio chemotherapy** **(durvalumab)** n= 5	–	26 weeks	**Pericardial effusion** ICI: n=2/63 (3.2%)
**Landman et al.** (2021) (19)	Israel	Retrospective study	66.5	**Total** n=39 **Male** n=25 **Female** n=14	**Smoking status** *Current* n=33 *Former/Never* n=6	**NSCLC** (72% ADK, 28% SCC)	Stage IIIA - IIIB	**Durvalumab following high dose radiotherapy** n=39	-	-	**Pericardial effusion** ICI: n=1/39
**Liu et al.** **(2022**) (20)	China	Prospective observational study	60.7	**Total** n=36 **Male** n=28 **Female** n=8	**Smoking status** *Current* n=18 *Former/Never* n=18 **Comorbidities** *Coronary artery disease* n=5 *Hypertension* n= 11 *Other related* n=7	NSCLC	-	**ICI (anti-PD-1/anti-PD-L1)** n= 36	-	12 weeks	**Cardiac dysfunction** **(LV-GRS)** ICI: n=7/36
**Moey et al.** **(2020**) (21)	USA	Retrospective observational study	**MACE group:** 64.3 **Non-MACE group:** 68.7	**Total** n=196 **Male** n=114 **Female** n=82	-	**NSCLC:** 179 **SCLC:** 18	III-IV	**ICI (anti-PD-1, anti-PD-L1/anti-PD-L1 + anti-CTLA-4)** n= 196	-	6.6 weeks	**MACE** n= 23/196 **Myocarditis** n=9/23 **NSTEMI** n=3/23 **SVT** n=7/23 **Pericardial disorders** n=4/23
**D’Souza et al.** **(2021**) (22)	Denmark	Retrospective cohort study	71	**Total** n=25573 **Male** n=12918 **Female** n=12655	**Comorbidities** *Hypertension* n=9511 *Myocardial infarction* n=1949 *Heart failure* n=1894 *Myocarditis* n=239 *Arrhythmia* n=3529 *Diabetes mellitus* n=3368	-	-	**ICI (anti-PD-1)** n= 743	**Non-ICI** n= 24830	**Cardiac event** 13.3 weeks **Arrhythmia** 19 weeks **Heart failure** 27.7 weeks **Peri- or myocarditis** 10.7 weeks **Cardiovascular death** 14.4 weeks	**Arrhythmia** n=27/743 **Heart failure** n=12/743 **Peri- or Myocarditis** n=11/743 **Cardiovascular death** n=18/743
**Faubry et al.** **(2022**) (23)	France	Prospective cohort study	64	**Total** n=99 **Male** n=51 **Female** n=48	**Smoking status** *Current* n=49 *Former* n=40 *Never* n=10 **Comorbidities** *Hypertension* n=18 *Coronary artery disease* n=14 *Arrhythmia* n=13 *Heart failure* n=15 *Diabetes mellitus* n=19 *Dyslipidaemia* n=32	**NSCLC:** 82 (66% ADK, 17% SCC) **SCLC:** 12 **Others:** 5	IIIB – IV	**ICI (single anti-PD-1/anti-PD-L1)** n=33 **Chemo+ICI** n=66	-	20.5 weeks	**Myocarditis** n=3/99 (1 case single agent and 2 cases reported combination agents).
**Isawa et al.** (2022) (24)	Japan	Prospective observational study	71	**Total** n=129 **Male** n=100 **Female** n=29	**-**	**NSCLC:** 107 (43% ADK, 39% SCC)	III – IV	**ICI (single anti-PD-1/anti-PD-L1)** n=129	-	**Abnormal laboratory findings (BNP elevation ≥200 pg/mL)** 18.3 weeks **Troponin T conversion** 8 weeks **ECG abnormal** 17.7 weeks **Myocarditis** 59.9 weeks **Heart failure** 18.3 weeks **CV-irAEs (ASCO)** *Grade ≥1* 10.3 weeks *Grade 1* 18.3 weeks *Grade ≥2* 20.1 weeks	**Abnormal laboratory findings (BNP elevation ≥200 pg/mL)** n=15/129 **Troponin T conversion** n=13/129 **ECG abnormalities** n=14/129 **Myocarditis** n=1/129 **Heart failure** n=6/129 **CV-irAEs (ASCO)** *Grade ≥1* n=35/129 *Grade 1* n=22/129 *Grade ≥2* n=13/129
**Jain et al.** (2021) (25)	USA	Retrospective cohort study	**ICI cohort:** 61 **Non-ICI cohort:** 65	**All tumor types** n=31659 **Male** n=16267 **Female** n=15392 **Lung cancer cohort:** n=9820	**Comorbidities** **ICI cohort (all tumor types):** *Hypertension* n=8033 *Myocardial infarction* n=664 *Heart failure* n=1340 *Diabetes mellitus* n=3099 **Non-ICI cohort (all tumor types):** *Hypertension* n=14357 *Myocardial infarction* n=1143 *Heart failure* n=2571 *Diabetes mellitus* n=7355	**Lung cancer + other tumors**	III – IV	**ICI cohort lung cancer (anti-PD-1, anti-PD-L1, anti-PD-L1/anti-PD-L1+ anti-CTLA-4)** n=5255	**Non-ICI cohort lung cancer** n=4565	**ICI-cohort:** **Stroke** 13.5 weeks **Heart failure** 16.4 weeks **Myocardial infarction** 15.5 weeks **Conduction disorder** 19.3 weeks **Non-ICI cohort:** **Stroke** 44.4 weeks **Atrial fibrillation** 29.4 weeks **Heart failure** 37.2 weeks **Conduction disorder** 43.4 weeks **Myocardial infarction** 35.4 weeks **Myocarditis** 9.28 weeks	**ICI cohort (LC):** **Stroke** n=184/5255 **Atrial fibrillation** n=184/5255 **Heart failure** n=205/5255 **Conduction disorder** n=66/5255 **Myocardial infarction** n=58/5255 **Myocarditis** n=3/5255 **Non-ICI cohort (LC):** **Myocardial Stroke** n=291/4565 **Atrial fibrillation** n=304/4565 **Heart failure** n=353/4565 **Conduction disorder** n=131/4565 **Myocardial infarction** n=102/4565 **Myocarditis** n=0/4565
**Iwai et al.** **(2023**) (26)	Japan	Retrospective cohort study	65	**Total** n=75807 **Male** n=55467 **Female** n=20340	-Before overlap weighting method- **ICI cohort:** **Smoking status:** *Current/Former* n=5155 *Never* n=1469 **Comorbidities:** *Hypertension* n=1079 *Dyslipidaemia* n=431 *Diabetes mellitus* n=959 *COPD* n=791 *Atrial* *fibrillation/flutter* n=54 **Non-ICI cohort** **Smoking status:** *Current/Former* n=44542 *Never* n=18923 **Comorbidities:** *Hypertension* n=13882 *Dyslipidaemia* n=5170 *Diabetes mellitus* n=10400 *COPD* n=10469 *Atrial* *fibrillation/flutter* n=807	**NSCLC:** 75807	III – IV	**ICI (single anti-PD-1/anti-PD-L1)** *Before overlap weighting method:* n=7177 *After overlap weighting* method: n=37903	**Non-ICI** *Before overlap weighting method:* n= 68630 *After overlap weighting method:* n=37903		**ICI cohort:** **VTEs** n=96/7177 **ATEs** n=38/7177 **Non-ICI cohort** **VTEs** n=665/68630 **ATEs** n=351/68630
**Khorana et al.** **(2023**) (9)	USA	Retrospective cohort study	62	**Total** n=2299 **Male** n=1274 **Female** n=1025	**ICI cohort:** n=605 *Hypertension* n=388 *Diabetes mellitus (complicated):* n=45 *Renal disease:* n=65 *COPD* n=393 *Atrial* *fibrillation/flutter* n=79 **Chemo cohort:** n=1092 *Hypertension* n=675 *Diabetes mellitus (complicated):* n=78 *Renal disease* n=82 *COPD* n=710 *Atrial* *fibrillation/flutter* n=109 **ICI + chemo cohort:** n=602 *Hypertension* n=371 *Diabetes mellitus (complicated)* n=39 *Renal disease* n=41 *COPD* n=395 *Atrial* *fibrillation/flutter* n=51	**NSCLC:** 2299	IV	**ICI cohort:** n=605	**Chemo cohort:** n=1092 **ICI + chemo cohort:** n=602	**ICI cohort:** 13.2 weeks **Chemo cohort:** 15.6 weeks **ICI + chemo cohort:** 11.6 weeks	**ICI cohort** *VTEs* n=81/605 **Chemo cohort:** *VTEs* n=197/1092 **ICI + chemo cohort** *VTEs* n=109/602
**Deschênes-Simard et al.** (2021) (27)	Canada	Retrospective cohort study	66.7	**Total** n=593 **Male** n=322 **Female** n=271	**Smoking status:** *Current* n=179 *Former* n=367 *Never* n=47 **Comorbidities:** *Hypertension* n=165 *Dyslipidaemia* n=128 *COPD* n=120 *Other cancer types* n=92 *Previous venous thrombosis* n=65 *Atrial* *fibrillation/flutter* n=29	**NSCLC:** 568 (95.7% ADK 4.3% SCC) **Other:** 25	II– IV	**ICI (single anti-PD-1/anti-PDL-1)** n=562 **ICI (≥2 immunotherapy agents)** n=31	-	15.2 weeks	**VTEs** n=59/593

ADK, adenocarcinoma; ATEs, arterial thrombotic events; ASCO, American Society of Clinical Oncology; BNP, B-type natriuretic peptide; COPD, chronic obstructive pulmonary disease; CTLA-4, cytotoxic T-lymphocyte-associated protein 4; CV, cardiovascular; CV-irAEs, cardiovascular immune-related adverse events; ECG: electrocardiogram; ICI, immune checkpoint inhibitor; ICSR, individual case safety report; LC, lung cancer; LV-GRS, left ventricular global radial stain; MACE, major adverse cardiac events; NSCLC, non-small cell lung cancer; NSTEMI, non-ST-segment elevated myocardial infarction; PD-1, programmed cell death protein 1; PD-L1, programmed death-ligand 1; SCC, squamous cell carcinoma; SCLC, small cell lung cancer; SVT, supraventricular tachycardia; VTEs, venous thrombotic events; WHO, World Health Organization.

(*) Comorbidity defined from registered diagnoses codes (hospitalizations or outpatient visits) within 5 years before index (22).

### 3.3 Publication bias and quality assessment

**Figure 2:**
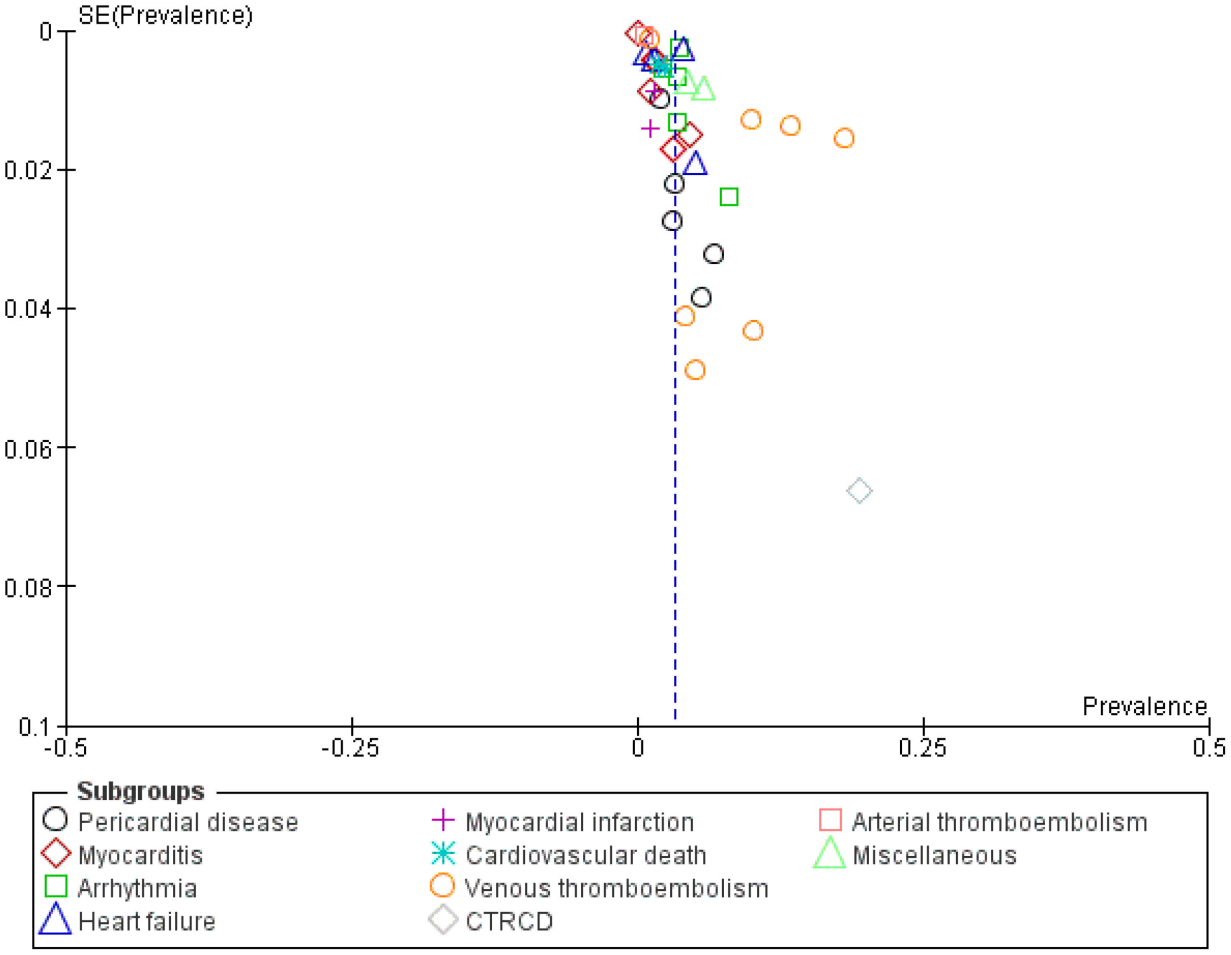
Funnel plot illustrating cardiovascular side effects in immune checkpoint Inhibitor-treated patients across included studies.

Publication bias was analyzed through a funnel plot (**Figure 2**), which indicated a symmetrical distribution, suggesting that no publication bias was present in the analysis. This plot is a graphical representation that displays the precision of the estimated treatment effect on the x-axis, and the sample size of each study on the y-axis. The presence of publication bias would have manifested as an asymmetrical plot, indicating that smaller studies with negative or null results were not being published. Therefore, the symmetrical distribution observed on our funnel plot indicated that there was no evidence of publication bias, providing an extra level of confidence in the validity of the study results.

Out of the twelve studies incorporated in our analysis, four demonstrated a moderated risk of bias, while the remaining eight displayed a low risk of bias, resulting in a cumulative score of 7.5, as shown in **Table 2**.

**Table 2:** Quality assessment of included studies.

STUDY	SELECTION				COMPARABILITY	OUTCOME			TOTAL SCORE
	Represent of exposed cohort	Selection of non- exposed cohort	Ascertainment of exposure	Outcome		Assessment of outcome	Length of follow-up	Adequacy of follow-up	
**Canale et al., 2020** (17)	1	1	1	0	2	1	1	1	8
**Moey et al., 2020** (21)	1	0	1	0	2	1	1	1	7
**Divisi et al., 2021** (18)	1	0	1	1	1	1	1	1	7
**Landman et al., 2021** (19)	1	0	1	1	2	1	1	1	8
**Jain et al., 2021** (25)	1	1	1	0	2	1	1	1	8
**Deschênes-Simard et al., 2021** (27)	1	1	1	0	2	1	1	1	8
**Liu et al., 2022** (20)	1	0	1	1	1	1	1	1	7
**D’souza et al., 2021** (22)	1	1	1	0	2	1	1	1	8
**Faubry et al., 2022** (23)	1	0	1	1	0	1	1	1	6
**Isawa et al., 2022** (24)	1	0	1	1	2	1	1	1	8
**Iwai et al., 2023** (26)	1	1	1	0	2	1	1	1	8
**Khorana et al., 2023** (9)	1	1	1	0	2	1	1	1	8

### 3.4 Meta-analysis results

**Figure 3:**
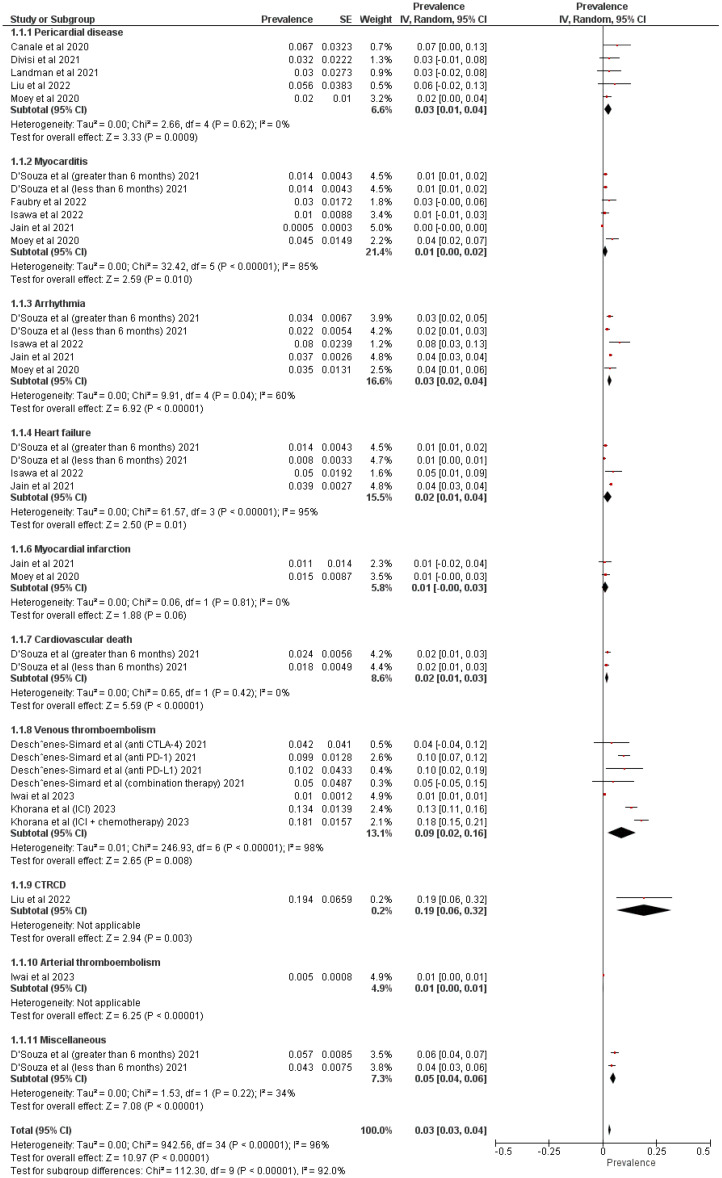
Forest Plot illustrating the prevalence of cardiovascular events in patients with lung cancer treated with immune checkpoint inhibitors.

**Figure 4:**
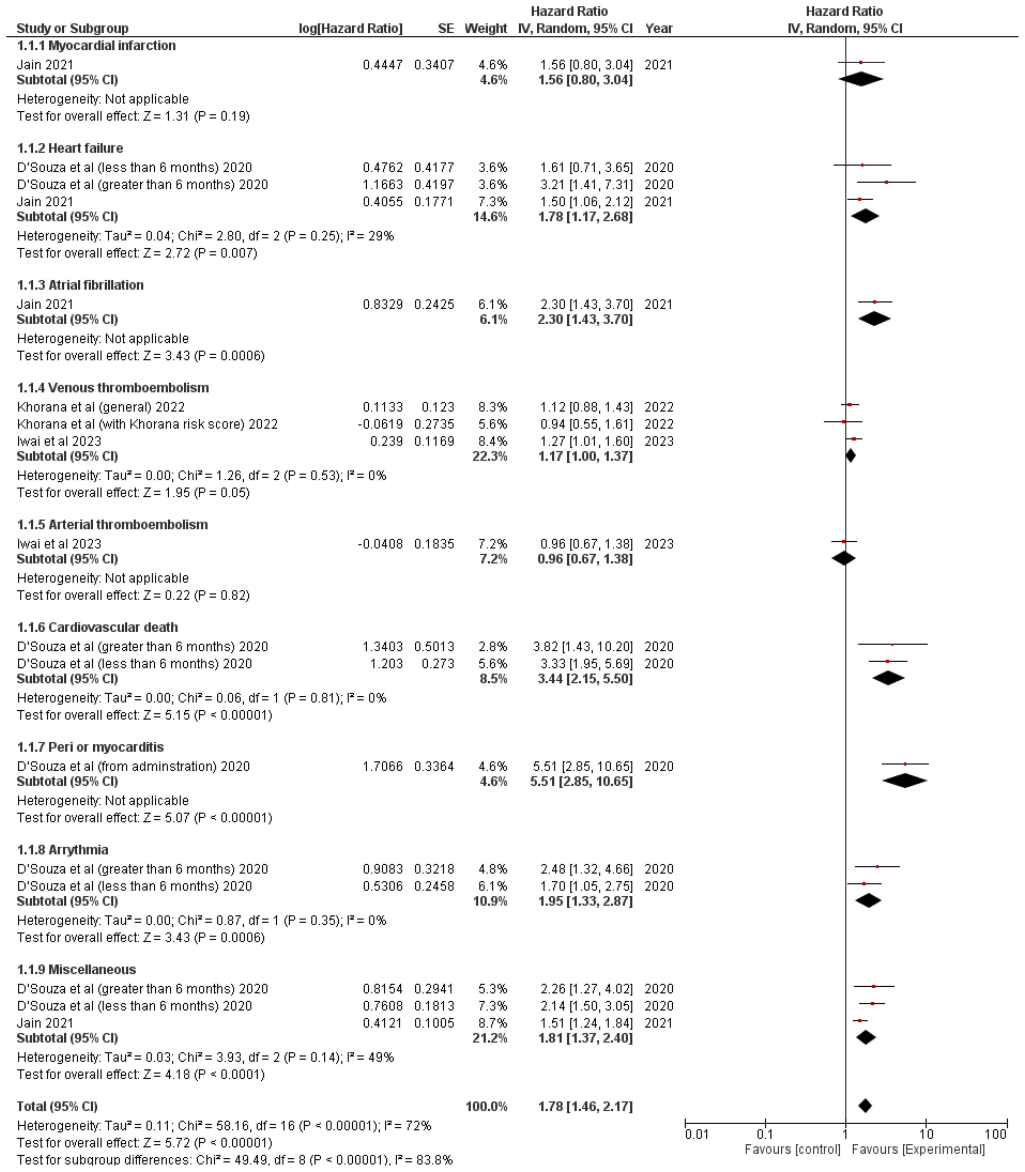
Forest Plot illustrating the hazard ratio in patients with lung cancer treated with immune checkpoint inhibitors.

Twelve cohort studies were used to assess the prevalence of CV events in patients with LC receiving ICI treatment. **Figures 3**, **4** show pooled results evaluating the prevalence and pooled HR.

#### 3.4.1 Prevalence of CV events

The following factors were evaluated in patients with LC: pericardial disease, myocarditis, arrhythmia, heart failure, venous thromboembolism, myocardial infarction, vasculitis, CV death, cancer therapy-related cardiac dysfunction (CTRCD), arterial thromboembolism, and miscellaneous. Five studies assessed pericardial disease (prevalence = 3% (95% CI: 1%-4%); p = 0.0009; I2 = 0%) (17–19, 21, 22) and myocarditis (prevalence = 1% (95% CI: 0%-2%); p = 0.01; I2 = 85%) (21–25), four studies estimated arrhythmia (prevalence = 3% (95% CI: 2%-4%); p < 0.00001; I2 = 60%) (21, 22, 24, 25) (9, 26, 27),, three studies analyzed heart failure (prevalence = 2% (95% CI: 1%-4%); p = 0.01; I2 = 95%) (22, 24, 25) and venous thromboembolism (prevalence = 9% (95% CI: 2%-16%); p = 0.008; I2 = 98%) (9, 26, 27), two studies calculated myocardial infarction (prevalence = 1% (95% CI: 1%-1%); p < 0.00001; I2 = 0%) (21, 25), and one study each assessed CV death (prevalence = 2% (95% CI: 1%-3%); p < 0.00001) (22), CTRCD (prevalence = 19% (95% CI: 6%-32%); p = 0.003) (20), arterial thromboembolism (prevalence = 1% (95% CI: 0%-1%); p < 0.00001) (26), and miscellaneous events (prevalence = 5% (95% CI: 4%-6%); p < 0.00001; I2 = 34%) (22) (**Figure 3**).

The overall appearance of CV events was statistically higher in patients who received ICI treatment compared to those who did not undergo ICI treatment (prevalence = 3% (95% CI: 3%-4%); p < 0.00001; I2 = 96%).

#### 3.4.2 HR of CV events

The following factors were assessed: heart failure, venous thromboembolism, miscellaneous, myocardial infarction, atrial fibrillation, arterial thromboembolism, CV death, peri- or myocarditis, and arrhythmia. Two studies assessed heart failure (HR = 1.78 (1.17, 2.68); p = 0.007; I2 = 29%) (22, 25), venous thromboembolism (HR = 1.17 (1, 1.37); p = 0.05; I2 = 0%) (9, 26), and miscellaneous events (HR = 1.81 (1.37, 2.4); p < 0.0001; I2 = 49%) (22, 25), and one study each was used to evaluate myocardial infarction (HR = 1.56 (0.8, 3.04); p = 0.19) (25), atrial fibrillation (HR = 2.3 (1.43, 3.7); p = 0.0006) (25), arterial thromboembolism (HR = 0.96 (0.67, 1.38); p = 0.82) (26), CV death (HR = 3.44 (2.15, 5.5); p < 0.00001; I2 = 0%) (22), peri- or myocarditis (HR = 5.51 (2.85, 10.65); p < 0.00001) (22), and arrhythmia (HR = 1.95 (1.33, 2.87); p = 0.0006; I2 = 0%) (22) (**Figure 4**).

The overall HR of CV events in lung cancer patients was statistically higher among patients treated with ICIs compared to those who did not receive ICI treatment HR: 1.78 (95% CI: 1.46, 2.17); p < 0.00001; I2 = 72%).

No significant differences were observed in HR or prevalence when studies one by one were removed from the analysis.

## 4 Discussion

Although the use of ICIs for the treatment of LC has demonstrated an improvement in outcomes of patients (5), this type of immunotherapy can also lead to a spectrum of CV complications, including pericardial disease and myocarditis, arrhythmia, heart failure, and VTE. Therefore, the main aim of this study was to improve comprehension of CV toxicity related to ICIs and to evaluate the prevalence and hazard ratios for various CV conditions.

Our study was consistent with previous systematic reviews and meta-analyses aimed at assessing the cardiac toxicity associated with ICIs and its worth highlighting that our meta-analysis is the first to incorporate observational studies that used RWD in non-selected population rather than solely relying on clinical trials data. Liu et al. conducted a meta-analysis based on 91 randomized controlled trials (RCTs) (n = 52,247), which found that the incidence of grade 1-5 CV toxicity and grade 3-5 CV toxicity was 3.23% and 0.97%, respectively. Additionally, ICI treatment increased the risk of CV toxicity compared to non-ICI therapy with a corresponding relative risk of 1.45 for grade 1-5 CV toxicity events and 1.55 for grade 3-5 CV toxicity events (28). Zhang et al. performed a meta-analysis of CV toxicity in lung cancer patients based on 38 RCTs (n = 14,342 patients) and found that adverse event (AE) risk ratios with a single ICI plus chemotherapy were 1.677-fold higher than with chemotherapy, which was statistically significant. However, no significant differences were found between single ICI and chemotherapy or single ICI and dual ICI combination therapy (29). In contrast, a meta-analysis by Jin et al. based on 17 RCTs (n = 11,063) evaluating ICI toxicity, has shown that CTLA + chemotherapy combination is associated to the lowest probability of CV toxicity, while dual ICI combination therapy (PDL-1 + CTLA-4) is associated to the highest probability of CV toxicity (30).

Regarding the prevalence of CV events in LC patients receiving ICI therapy, our findings were not negligible, with 3% of events and a HR of 1.78. The risk of developing CV immune-related adverse events (CV-irAEs) was increased in patients undergoing combination therapy. Among the various CV-irAEs, pericardial disease and myocarditis stand out with prevalence rates of 3% and 1%, respectively. Patients treated with ICIs exhibited a more than 5-fold higher risk of developing pericarditis or myocarditis (HR = 5.51 [2.85-10.65, p < 0.001]). In accordance with our findings, a retrospective study conducted at a single academic center found a more than 4-fold increase in pericarditis or pericardial effusion incidence in patients receiving ICI compared to control subjects (31). Additionally, an increased prevalence of cardiac arrhythmias, heart failure, and VTE in patients undergoing ICI therapy was also observed, ranging from 2% to 3% (31). A study by Kondapalli et al. involved a cohort of 1,813 patients treated with ICI with a mean follow-up of 4.6 ± 3.4 years (3.2 ± 3.2 years pre-ICI and 1.4 ± 1.4 years post-ICI). VTEs dominated as the most common cardiovascular complication, affecting 11.4% of patients both before and after ICI therapy. Following treatment, 3.0% of patients experienced a myocardial infarction, 2.8% developed heart failure, and 1.6% suffered a stroke (32).

CV toxicity risk stratification, along with biomarker surveillance and innovative cardiac imaging parameters have enhanced the ability to predict CV toxicity. However, there are inconsistencies in the frequency and timing of cardiac troponin (cTn) measurements across different studies. For instance, Puzanov et al. recommended that the measurement of cTn levels before starting treatment and at regular intervals, which may vary between two weeks and three months after treatment (33). Other researchers recommended regularly checking cTn values weekly during the first six weeks of treatment, in addition to assessing other biomarkers and performing ECG tests (34). The 2022 ESC Guideline on cardio-oncology recommend baseline cTn measurement in patients with an indication for ICI treatment (Class I) (35). Nevertheless, American Society of Clinical Oncology (ASCO) guidelines strongly discourage the use of cardiac biomarker testing in patients undergoing ICI treatment, since there is no clear evidence regarding the efficacy or value of routine baseline or serial electrocardiograms (ECGs) or cTn measurements in patients receiving ICIs (36). It is only advisable to perform an ECG before therapy and continuously monitor cTn levels when patients are undergoing combination immunological treatment. Based on the signs and symptoms observed, additional testing may be performed, including echocardiography, assessment of natriuretic peptide levels, and stress testing. Additionally, Moslehi et al. recommended serial echocardiographic screening for high-risk patients, including those with pre-existing cardiac disease, combined ICI, or other drugs with known CV toxicity (37).

Magnetic resonance imagining (MRI) is crucial for diagnosing myocarditis because it assesses the presence of increased blood flow, swelling, and tissue death in the myocardium. Hyperemia is detected by early gadolinium enhancement (EGE), which reveals a rapid uptake of contrast medium due to increased permeability of blood vessels and cellular death. Edema is identified using T2-weighted sequences that highlight regions with increased water content. Necrosis can be observed with late gadolinium enhancement (LGE), which shows strong signals in areas of necrotic tissue following contrast administration. Advanced T1 and T2 mapping techniques enable accurate quantification of tissue properties, improving diagnostic precision. Nevertheless, the true prevalence of myocarditis may be underestimated due to the financial and logistical challenges associated with using these sophisticated imaging methods (38).

To address this issue, the Spanish Immunotherapy Registry of Cardiovascular Toxicity (SIR-CVT) have initiated a registry with the aim of identifying the risk factors associated with ICI-induced cardiovascular toxicity, to optimize its monitoring, and to anticipate its possible adverse events (39).

Hence, this meta-analysis suggests that immunotherapy is associated with CV toxicity in RWD similar to what it has been reported in selected patients included in clinical trials. However, most clinical trials and routine clinical practice did not include systematic cardiac monitoring, complicating the ability to identify CV toxicity. In addition, the majority of reported treatments consisted of a combination of multiple anticancer drugs, making it difficult to determine the specific agent responsible for CV toxicity or whether a particular drug has a greater propensity for CV toxicity. Patients using ICIs still require regular monitoring of cardiac function in the clinic, including cTn, ECG, and cardiac ultrasound. When selecting immunotherapies and combination therapies, it is important to consider the patient’s genetic and tumor-specific variables to prevent resistance and adverse outcomes associated with these treatments. Moreover, oncologists should collaborate closely with cardiologists to ensure optimal management of cardiac health throughout the course of immunotherapy. This collaborative approach should emphasize the preventive role of cardio-oncologists, starting with a baseline evaluation where all risk factors are identified and aggressively treated. Preventive efforts must also include promoting healthy lifestyle behaviors and continue throughout and after oncologic treatment. Notably, a significant drop in mortality rates, particularly for ICI-myocarditis, has been observed over the last decade. This improvement likely reflects better recognition of this disease, including smoldering non-fulminant cases, and advances in appropriate therapeutic management (40).

While myocarditis has been recognized as a primary cardiac adverse event, emerging evidence underscores the impact of ICI therapy on the atherosclerotic pathway, which warrants further exploration. Recent studies suggest that ICIs exacerbate systemic inflammation—a key driver of atherosclerosis—thereby accelerating the progression and increasing the vulnerability of atherosclerotic plaques. Preclinical research has demonstrated that ICI therapies, particularly those targeting PD-1 and CTLA-4, induce T-cell–mediated plaque inflammation, enlarge necrotic core size by 3.9-fold, and promote vascular endothelial activation by 2.2-fold, highlighting the role of short-term ICI therapy in driving plaque progression through T-cell–mediated inflammation (41).

Furthermore, observational studies have consistently reported a 3- to 7-fold increase in CV events following the initiation of ICIs, with accelerated non-calcified plaque progression being particularly evident in patients with lung cancer (42, 43). Although the pathophysiological mechanisms underlying ICI-induced atherosclerosis remain incompletely understood, studies suggest these effects are likely associated with inflammation and immune dysregulation (44).

Nonetheless, preclinical studies have yet to fully elucidate how these alterations affect the various stages of atherosclerosis. It is increasingly evident that the microenvironmental context of cell death and apoptosis plays a critical role in determining whether ICIs exhibit atherogenic or atheroprotective effects. Consequently, the impact of ICIs on atherosclerosis may vary depending on the stage of disease progression. Further mechanistic studies are essential to better understand these effects and inform the timing and nature of potential interventions. Clinically, the link between ICIs and atherosclerosis has primarily been established through smaller observational studies, emphasizing the need for larger, long-term investigations to confirm this association and quantify the incidence of adverse cardiovascular events (45).

Our study had the following limitations: (a) only observational studies were included; (b) moderate heterogeneity was observed in HR while high heterogeneity was seen in prevalence analysis; (c) only 4 studies were used in analyzing HR. Nonetheless, these studies were pivotal in performing our analysis and conducting this research.

## 5 Conclusions

The meta-analysis involving 23,621 lung cancer patients revealed a 3% prevalence of cardiac events among those treated with ICIs, with a hazard ratio of 1.78. Moreover, the incidence of cardiovascular events in patients with LC was significantly higher in those who received ICI treatment compared to those who did not. This study confirms the findings of previous research, reinforcing the importance of understanding the relationship between cancer treatments and cardiac health. By cross-referencing results from different studies, a more extensive comprehension of CV toxicity patterns can be achieved, thus enhancing the reliability of the conclusions drawn. The complexities of CV toxicity require a cohesive effort to balance treatment efficacy with cardiac safety. Moreover, this meta-analysis sheds light on the different risks associated with these treatments, guiding clinicians to make informed decisions that prioritize both cancer control and cardiac health.

## Data Availability

The original contributions presented in the study are included in the article/supplementary material. Further inquiries can be directed to the corresponding author.
